# Complete Genome Sequence of *Streptococcus pyogenes emm14* JS95, a Necrotizing Fasciitis Strain Isolated in Israel

**DOI:** 10.1128/genomeA.00025-17

**Published:** 2017-03-16

**Authors:** Jacqueline L. Y. Chee, Miriam Ravins, Emanuel Hanski, Swaine L. Chen

**Affiliations:** a Genome Institute of Singapore, Singapore; b Hebrew University of Jerusalem, Jerusalem, Israel; c Singapore-HUJ Alliance for Research and Enterprise, Singapore; d National University of Singapore, Singapore

## Abstract

Here, we report the complete genome sequence of the *Streptococcus pyogenes emm14* strain JS95, isolated from a patient with necrotizing fasciitis. The streptococcal invasion locus (*sil*), the first quorum-sensing system characterized in *S. pyogenes*, was identified in this strain.

## GENOME ANNOUNCEMENT

*Streptococcus pyogenes*, or group A streptococcus (GAS), is an important Gram-positive human pathogen that causes a wide variety of infectious diseases ranging from benign to severe infections, such as necrotizing fasciitis (NF) (1). Here, we report the whole-genome sequence of the GAS *emm14* strain JS95, isolated from an NF patient during a prospective nationwide, population-based study of invasive GAS infections conducted in Israel (2). JS95 is capable of generating an NF-like lethal invasive infection in a murine model of human soft tissue infection (3, 4). By applying transposon-tagged mutagenesis on JS95 and using a murine model, the streptococcal invasion locus (*sil*) was initially identified (4). The quorum-sensing (QS) module of *sil* was further characterized, and it was shown that, by restoring the start codon by mutating an ATA to ATG in the autoinducer peptide, QS became fully active, in that JS95 could now both sense and produce the autoinducer peptide SilCR (5–7). JS95 has been utilized in the past 20 years for the study of GAS virulence. *In vivo* and *in vitro* experiments on JS95 led to the finding that ScpC is the GAS serine protease responsible for cleavage and inactivation of both human (IL-8) and mouse (KC and MIP-2) CXC PMN chemokines (8). Recently, it was demonstrated that GAS induces endoplasmic reticulum stress and the unfolded protein response through which it captures the amino acid asparagine from the host that is utilized for regulating its own sensing and proliferation (5).

JS95 genomic DNA was sheared to approximately 300 bp using a focused ultrasonicator (Covaris). A sequencing library was prepared using the TruSeq DNA library prep kit (Illumina) according to the manufacturer’s instructions. This was sequenced using an Illumina HiSeq2000 platform with 2 × 76-bp reads. The data were assembled using Velvet version 1.2.10 (9) with a minimum contig cutoff of 500 bp, scaffolded with OPERA version 1.4.1 (10), and finished with FinIS version 0.3 (11). In total, 10,892,506 paired-end reads passed filtering, representing an approximate coverage of 900× (based on the final assembly).

The JS95 assembly consists of 30 contigs, which range in size from 639 to 269,693 bp. The combined length of all contigs is 1,784,810 bp, with an *N*_50_ size of 126,582 bp and a G+C content of 38.4%. Annotation of the draft assembly was performed using the NCBI Prokaryotic Genome Annotation Pipeline (PGAAP) (12), which predicted 1,725 protein-coding sequences and 38 tRNA genes.

### Accession number(s).

The *S. pyogenes* JS95 draft assembly has been deposited at DDBJ/ENA/GenBank under the accession number MSMN00000000. The version described in this paper is the first version, MSMN01000000. The raw Illumina reads have been deposited in GenBank under BioProject PRJNA358871.
